# Exenatide Treatment Causes Suppression of Serum Ghrelin Levels following Mixed Meal Test in Obese Diabetic Women

**DOI:** 10.1155/2016/1309502

**Published:** 2016-02-22

**Authors:** Figen Topyildiz, Sinem Kiyici, Zulfiye Gul, Deniz Sigirli, Metin Guclu, Gurcan Kisakol, Sinan Cavun

**Affiliations:** ^1^Bursa Yuksek Ihtisas Education and Research Hospital, Department of Internal Medicine, 16330 Bursa, Turkey; ^2^Uludag University Medical Faculty, Department of Pharmacology, 16059 Bursa, Turkey; ^3^Uludag University Medical Faculty, Department of Bio-Statistics, 16059 Bursa, Turkey; ^4^Bursa Yuksek Ihtisas Education and Research Hospital, Department of Endocrinology and Metabolism, 16330 Bursa, Turkey

## Abstract

*Aim*. To investigate the effect of exenatide treatment on serum ghrelin levels in obese female patients with type 2 diabetes mellitus.* Methods*. Fourteen female patients with type 2 diabetes mellitus being treated with metformin and exenatide were enrolled. A mixed meal test was applied to the patients while continuing with their daily medications. Blood samples were taken before and at 60, 120, and 180 minutes following mixed meal test to measure serum total ghrelin, glucose, and insulin levels. The following week, exenatide treatment of the patients was paused for 24 hours and the same experimental procedures were repeated.* Results*. Serum ghrelin levels were suppressed significantly at 180 minutes with exenatide treatment compared with baseline (294.4 ± 57.5 versus 234.5 ± 59.4 pg/mL) (*p* < 0.001). Serum ghrelin levels at 180 minutes were statistically different when percentage change in serum ghrelin levels after mixed meal tests with and without exenatide usage were compared (*p* = 0.001). Estimated total area under the curve values for serum ghrelin concentrations was also significantly lower with exenatide compared with omitted treatment (*p* = 0.035).* Conclusion*. These results suggest that the effect of exenatide on weight loss may be related with the suppression of serum ghrelin levels, which is an orexigenic peptide.

## 1. Introduction

Ghrelin is a potent gut-brain orexigenic peptide produced by enteroendocrine cells within the mucosal layer of the stomach. Ghrelin plays an important role in the stimulation of food intake and long-term regulation of body weight [1, 2]. Plasma ghrelin concentrations increase with fasting and decrease postprandially, suggesting a physiological role for ghrelin in meal initiation in humans [3].

Weight gain is a significant problem in the treatment of patients with type 2 diabetes mellitus [4]. Excessive energy intake is one of the contributing factors which play a part in the observed weight gain in patients with type 2 diabetes [5]. Effective interventions designed to achieve weight reduction are a critical part of type 2 diabetic patients' management to prevent the development of chronic complications. However, many antidiabetic agents including insulin, sulfonylureas, and thiazolidinediones cause weight gain instead of weight loss during the management period [6]. Exenatide, a long acting glucagon-like peptide-1 (GLP-1) receptor agonist, is used in the treatment of patients with type 2 diabetes mellitus. Exenatide is a potential regulator of feeding behavior through its ability to inhibit gastric emptying, reduce food intake, and induce satiety. Weight loss with considerable variation has been reported in patients with type 2 diabetes mellitus during the exenatide treatment [7, 8]. The mechanism of how exenatide promotes weight loss is not clearly understood.

In a recent study it was reported that exenatide reduced the serum levels of ghrelin by up to 74% in fasting rats [9]. Patel et al. [10] also found that combined treatment of omeprazole with exendin-4 reduced food intake and body weight gain, most likely through changes in plasma ghrelin and leptin levels in rats. There is no study evaluating the effect of exenatide treatment on serum ghrelin levels in patients with type 2 diabetes mellitus. Taking the effects of ghrelin on food intake and body weight into consideration, ghrelin might be one of the contributors in the weight loss effect of exenatide treatment.

In this study, we aimed to investigate the effect of exenatide treatment on serum ghrelin levels following mixed meal test in obese female patients with type 2 diabetes mellitus.

## 2. Material and Methods

### 2.1. Subjects

This study was planned as a prospective cross-sectional trial. Patients were recruited from prescreened female patients with type 2 diabetes mellitus from an Endocrinology Outpatient Clinic of a tertiary referral center. Fourteen patients treated with metformin 1000 mg and exenatide 10 *µ*g per dose administered twice daily for at least 3 months were enrolled in the study. Body mass index (BMI) < 40 kg/m^2^ and 6.5% (48 mmol/mol) < hemoglobin A1c (HbA1c) < 8.5% (69 mmol/mol) were the additional inclusion criteria. Exclusion criteria included usage of any medications other than the study drugs and presence of cardiovascular, gastrointestinal, hepatic, renal, rheumatological, neoplastic, infectious, and other endocrine diseases, with the exception of type 2 diabetes mellitus. All patients were clinically free from micro- and macrovascular complications of diabetes and were nonsmokers. None of the patients had a previous history of substance abuse. Informed consent was obtained from all participants, and the study was performed in accordance with the Declaration of Helsinki and with the approval of the local ethics committee. Patients presented to the investigation center in the morning following a 12-hour fasting period. Body weight and height were measured in the fasting state. BMI was computed as weight in kilograms divided by height in meters squared. An intravenous catheter was inserted into an antecubital vein. Subsequently, patients received a standard mixed meal of 600 kcal, consisting of 50% carbohydrate, 20% protein, and 30% fat which was consumed over a 15-minute period. Metformin and exenatide treatments were administered 15 minutes before the consumption of the mixed meal in accordance with the patients routine. Venous blood samples were taken before (0 minutes) and at 60, 120, and 180 minutes following mixed meal test for the measurement of serum total ghrelin, glucose, and insulin levels. Insulin, glucose, and total ghrelin levels were measured at all time intervals. In the following week, the patients' exenatide treatment was paused for 24 hours while continuing to take metformin treatment. Mixed meal test was performed as described above and the same experimental procedures were repeated. Patients were observed in both test periods to evaluate whether they had consumed all the mixed meal. Metformin and exenatide treatments were well tolerated by the patients and we did not observe any side effects. Baseline biochemical parameters and HbA1c measurements were assessed from blood samples taken at zero point.

### 2.2. Measurements

Blood was collected into serum separator tubes with clot activator. Samples were centrifuged at 3000 g for 15 min to separate serum. All specimens were stored at −80°C until analysis. Serum total ghrelin levels were measured with the commercially available ELISA kit (RayBio Human/Mouse/Rat Ghrelin Enzyme Immunoassay, RayBiotech, Norcross, GA, USA). The sensitivity limit of total ghrelin was 0.1 ng/mL. Serum glucose levels were measured using the autoanalyzer system (Aeroset System Abbott, Abbott Laboratories, Diagnostic Division, IL, USA) and HbA1c levels were determined by high performance liquid chromatography (Trinity Biotech, Kansas City, USA). Serum insulin levels were measured by means of ADVIA Centaur Chemiluminescence Enzyme Immunoassay (Siemens Healthcare Diagnostics, NY, USA) (for SI unit conversions: mg/dL × 0.0555 mmol/L for glucose, *μ*IU/mL × 6.945 = pmol/L for insulin, and pg/mL × 0.3 = pmol/L for ghrelin).

### 2.3. Statistical Analyses

The data were analyzed using SPSS for Windows, version 21.0. Data were shown as mean ± standard deviation. A two-sided *p* value of less than 0.05 was considered statistically significant. Two-way repeated measures analysis of variance (ANOVA) was carried out. This analysis provides *p* values for the overall differences between the experiments (A), for differences over time (B), and for the interaction of experiment with time (AB). If a significant interaction of treatment and time (AB) was documented (*p* < 0.05), individual variables were compared by paired *t* test for normally distributed variables and Wilcoxon signed-rank test for nonnormal variables. Bonferroni correction was used after these univariate tests. Percentage changes were calculated as (60th minutes-baseline)/baseline, (120th minutes-baseline)/baseline, and so forth. Pearson correlation analysis was used to investigate association between serum ghrelin and the other laboratory parameters.

## 3. Results

Baseline characteristics and fasting metabolic parameters of all subjects are summarized in Table 1. Fourteen obese female patients without chronic micro- or macrovascular complications entered and completed the study. Mean age of patients was 51.7 ± 10.1 years with mean diabetes duration of 69 ± 12 months. Mean BMI was 37.0 ± 20.2 kg/m^2^ with mean waist circumference of 111.4 ± 8.2 cm. Mean HbA1c of patients was 7.4 ± 0.8% (57.3 ± 6.1 mmol/mol).

Two-way repeated measures ANOVA demonstrated that there was a significant effect of time (*p* < 0.001), treatment (*p* < 0.001), and treatment × time interaction effect (*p* = 0.001) for serum glucose. Also, there was a significant effect of time (*p* < 0.001), treatment (*p* = 0.002), and treatment × time interaction effect (*p* = 0.009) for insulin levels. For ghrelin levels, there was a significant effect of treatment (0.028) and treatment × time interaction (0.034), but no effect of time (*p* = 0.278).

**Figure 1:**
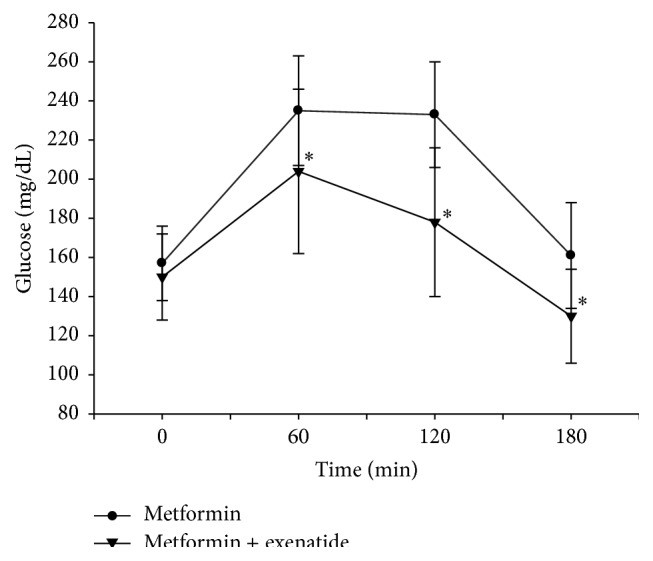
Serum glucose levels following mixed meal test with and without exenatide usage. ^*∗*^ *p* < 0.05 when the percentage changes in serum glucose levels of two treatments were compared.

**Figure 2:**
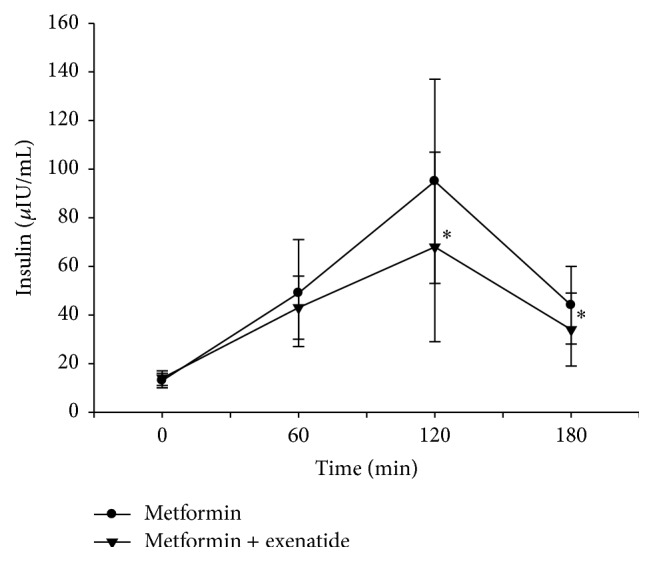
Serum insulin levels following mixed meal test with and without exenatide usage. ^*∗*^ *p* < 0.05 when the percentage changes in serum insulin levels of two treatments were compared.

Serum glucose concentrations peaked at 60 minutes and insulin levels at 120 minutes following mixed meal test with and without exenatide treatment (Table 2). Percentage changes in serum glucose levels at 60, 120, and 180 minutes after the mixed meal test were found significantly different with exenatide treatment when compared with the omitted exenatide treatment (*p* = 0.032, *p* < 0.001, and *p* = 0.005, resp.) (Figure 1) (Table 3). Percentage changes in serum insulin levels after mixed meal test were also significantly different with and without exenatide treatment at 120 and 180 minutes (*p* = 0.001 and *p* = 0.027, resp.). Postprandial serum insulin levels were lower with exenatide treatment compared with skipped exenatide treatment at all time intervals except at 60 minutes (Figure 2) (Table 3).

**Figure 3:**
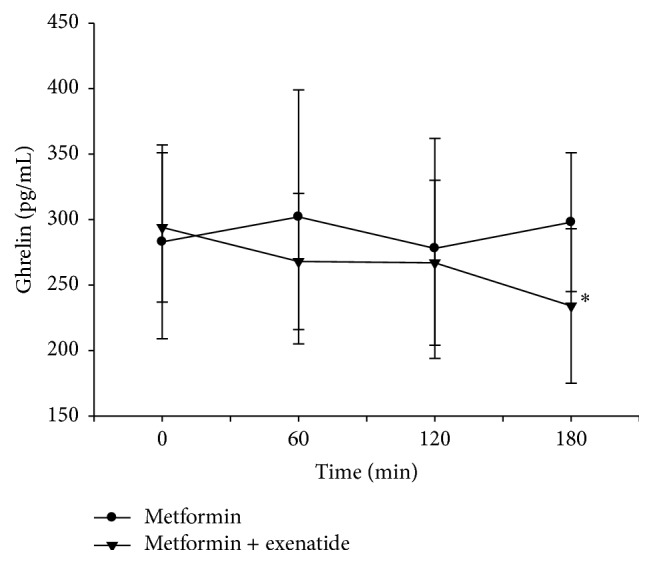
Serum ghrelin levels following mixed meal test with and without exenatide usage. ^*∗*^ *p* < 0.05 when the percentage changes in serum ghrelin levels of two treatments were compared.

Serum ghrelin levels were suppressed significantly at 180 minutes compared with baseline values after mixed meal test with exenatide treatment (294.4 ± 57.5 versus 234.5 ± 59.4 pg/mL) (*p* < 0.001) (Table 2). While percentage changes in serum ghrelin levels after mixed meal tests with and without exenatide usage were compared, no significant difference was found at the 60 and 120 minutes. However, percent changes in serum ghrelin levels at 180 minutes were statistically significant (−0.20 ± 0.15 versus 0.10 ± 0.29) (*p* = 0.001) and ghrelin levels were found to be suppressed with exenatide treatment (Table 3) (Figure 3).

Total area under the curve (AUC) was also calculated using the trapezoidal rule for serum glucose, serum insulin, and serum ghrelin concentrations, in patients with and without exenatide treatment. AUC values for serum glucose, insulin, and ghrelin concentrations were significantly lower with exenatide treatment compared with omitted exenatide treatment (*p* = 0.001, *p* = 0.004, and *p* = 0.035, resp.) (Table 4).

Serum ghrelin levels were not found correlated with serum insulin or glucose levels at any time interval with and without exenatide treatment according to the Pearson correlation analysis.

## 4. Discussion

In this study we observed that exenatide treatment causes suppression of serum ghrelin levels following mixed meal test in obese female patients with type 2 diabetes. GLP-1 receptor agonists, such as exenatide, are glucose-lowering drugs that mimic the action of an endogenous GLP-1. GLP-1 increases the first phase of insulin secretion and suppresses glucagon secretion and it is effective in improving glyceamic control [7, 11]. GLP-1 agonists cause weight reduction, in contrast to the weight gain seen with sulphonylureas, the glitazones, and insulin, and the weight neutral effects of gliptins [5, 6]. The most commonly reported adverse events were nausea, vomiting, and diarrhea. Gastrointestinal side effects were worst at the beginning and tended to reduce over the course of treatment [8]. Weight loss seen during the treatment is considered to be associated with the gastrointestinal side effects observed at the beginning of treatment. But several trials reported that weight loss also occurred in patients not experiencing nausea [12, 13]. Exactly how exenatide promotes weight loss is not clearly understood. Our findings suggest that ghrelin suppression induced by exenatide treatment could be playing a role in the weight loss effect of exenatide.

Ghrelin is a potent orexigenic and adipogenic peptide. Acute and chronic nutritional status change endogenous ghrelin secretion. Fasting causes elevation in serum ghrelin levels, which reduce 60–120 min after food intake, supporting a role for this peptide as a peripheral sensor of short-time positive energy imbalance [2, 3, 14]. Ghrelin also increases gastric acid secretion and motility [15, 16]. Ghrelin and GLP-1 have opposing effects on the gastrointestinal system when compared with each other. GLP-1 reduces gastric acid secretion and gastric emptying [17, 18]. Short term infusion of GLP-1 is associated with diminished appetite and reduced energy consumption in humans [19]. An inverse relationship between GLP-1 and ghrelin levels has been demonstrated after a standard glucose tolerance test in humans [20]. As far as we know there is no study evaluating the effect of exenatide treatment on serum ghrelin levels in patients with type 2 diabetes mellitus. However, there are some animal studies investigating the effect of exenatide on serum ghrelin levels. Patel et al. [10] found that combined treatment of omeprazole with exenatide reduces food intake and body weight gain, most likely through changes in plasma ghrelin and leptin levels in rats. Pérez-Tilve et al. [9] also reported that intraperitoneal and intracerebroventricular administered exenatide reduced the serum ghrelin levels and the food intake in a dose-dependent manner in fasting rats. They showed that the levels of ghrelin start to drop after 30 minutes and remained low for at least 8 hours. Insulin levels also remained low for up to 8 hours following exenatide administration. Consistent with these results, we found that exenatide treatment suppressed serum ghrelin levels following mixed meal test while there was no statistically significant change in serum ghrelin levels without exenatide. Mean percentage change in serum ghrelin levels at 120 minutes without exenatide treatment was −0.04 ± 0.51 and despite a decline in serum ghrelin levels from baseline to 120 minutes, it did not reach statistical significance. Increasing the number of patients and additional blood sampling at 90 and 150 minutes might yield more significantly statistical results. Moreover, it has been reported that fasting total ghrelin levels are lower in type 2 diabetic patients than healthy individuals. Serum ghrelin level reduction is also significantly less pronounced in patients with type 2 diabetes [21]. This might be another explanation of statistically insignificant postprandial insulin suppression in diabetic patients without exenatide treatment in our study.

Dipeptidyl peptidase-4 (DPP-4) inhibitors such as sitagliptin are effective in glucose metabolism by preventing DPP-4 from deactivating endogenous GLP-1 [22]. It has been reported that fasting serum ghrelin levels of patients with type 2 diabetes mellitus were significantly decreased after 12 weeks of sitagliptin treatment [23]. Patients in the present study were receiving both exenatide and metformin treatments. Metformin usage causes weight loss but the results of the studies investigating the effect of metformin treatment on serum ghrelin levels are conflicting [24, 25]. Suppression of serum ghrelin levels could not be attributed to metformin in our study because it was administered continually throughout the study. Together these data suggest that serum ghrelin suppression with exenatide usage in this study appears to be related with GLP-1 associated mechanisms.

Prader-Willi syndrome (PWS) is associated with hyperphagia and obesity. PWS has high circulating ghrelin levels that do not decline after a meal [26]. Sze et al. [27] investigated the effectiveness of exenatide in adult PWS subjects compared with obese controls. They reported that a single subcutaneous injection of 10 *μ*g exenatide increased satiety and lowered glucose and insulin levels but increased insulin secretion rate in both groups. They also found that exenatide treatment decreased PYY and glucagon-like peptide-1, whereas ghrelin levels remained unchanged in contrast to our results. However Senda et al. [28] reported that the GLP-1 receptor antagonist liraglutide suppresses ghrelin and controls diabetes in patients with PWS. Endogenous GLP-1 secretion is decreased in patients with type 2 diabetes mellitus [29] and for this reason GLP-1 receptor agonists treatment might have more effect on serum ghrelin levels compared with normal subjects. Postprandial serum glucose and insulin levels were lower with exenatide treatment compared with omitted exenatide treatment at all time intervals in our study as expected and in accordance with the literature [12, 13].

Hagemann et al. [30] reported that a GLP-1 infusion suppressed ghrelin levels and increased insulin in healthy male volunteers. They concluded that the ghrelin reduction was a likely consequence of elevated insulin levels. Previous studies indicate that insulin and hyperglycemia suppress circulating ghrelin levels [31, 32]. When the effects of exenatide administration on serum glucose and insulin levels are taken into consideration, the observed serum ghrelin suppression in the present study might be secondary to the mentioned hormonal changes induced by exenatide. However, in our study, serum glucose and insulin levels were significantly lower while serum ghrelin levels were more suppressed with exenatide treatment compared with omitted exenatide. These findings might suggest that there is also a direct interaction between exenatide treatment and ghrelin secretion. Delayed gastric emptying associated with exenatide treatment [33] might be another possible mechanism which contributes to the postprandial decline in serum ghrelin levels.

There are several limitations to our study. Our study population was small in order to draw a definitive conclusion with regard to the general population and consisted only of obese female diabetic patients. In addition, time intervals for the serum ghrelin measurements could be increased and prolonged to reach more statistically significant results.

In conclusion, these results suggest that exenatide treatment suppresses postprandial serum ghrelin levels in obese female patients with type 2 diabetes mellitus. Further studies are needed to clarify the exact effect of exenatide and the other GLP-1 receptor agonists treatment on serum ghrelin levels in order to confirm our findings.

## Figures and Tables

**Table 1:** Demographic characteristics and laboratory parameters of study patients.

Number of patients (*n*)	14
Age (years)	51.7 ± 10.1
Weight (kg)	101.0 ± 20.2
Body mass index (kg/m^2^)	37.0 ± 20.2
Waist circumference (cm)	111.4 ± 8.2
Hip circumference (cm)	128.7 ± 12.1
Body fat ratio (%)	43.7 ± 4.8
Systolic blood pressure (mmHg)	126.8 ± 12.7
Diastolic blood pressure (mmHg)	79.5 ± 9.0
HbA1c (%)	7.4 ± 0.8
Total cholesterol (mg/dL)	210 ± 39.7
HDL cholesterol (mg/dL)	51.9 ± 12.9
Triglyceride (mg/dL)	169.9 ± 34.3
LDL cholesterol (mg/dL)	123.6 ± 38.0
Creatinine (mg/dL)	0.8 ± 0.1
AST (U/L)	20.7 ± 10.7
ALT (U/L)	24.2 ± 12.0
TSH (*μ*IU/mL)	1.7 ± 1.0

Variables given as mean ± standard deviation.

**Table 2:** Serum glucose, insulin, and ghrelin levels before (0 minutes) and at 60, 120, and 180 minutes following mixed meal test with and without exenatide usage.

	Time (minute)	With exenatide (mean ± SD)	Without exenatide (mean ± SD)
Glucose (mg/dL)	0	150.0 ± 22.1	157.2 ± 19.7
	60	204.8 ± 42.0^*∗*^	235.8 ± 28.2^*∗*^
	120	178.2 ± 38.4^*∗*^	233.7 ± 27.5^*∗*^
	180	130.5 ± 24.3	161.7 ± 27.9

Insulin (*µ*g/mL)	0	14.2 ± 3.91	13.6 ± 3.1
	60	43.0 ± 13.8^*∗*^	49.9 ± 22.2^*∗*^
	120	68.8 ± 39.2^*∗*^	95.9 ± 42.5^*∗*^
	180	34.3 ± 15.5^*∗*^	44.6 ± 16.4^*∗*^

Ghrelin (pg/mL)	0	294.4 ± 57.5	283.7 ± 74.0
	60	268.2 ± 52.2	302.0 ± 97.3
	120	267.5 ± 63.3	278.7 ± 84.0
	180	234.5 ± 59.4^*∗*^	298.1 ± 53.1

SD: standard deviation. ^*∗*^Significant change compared to baseline value after Bonferroni correction (*α* ^*∗*^ = 0.017).

**Table 3:** Percentage changes in serum glucose, insulin, and ghrelin levels at 60, 120, and 180 minutes following mixed meal test with and without exenatide usage.

Percentage change (%)	Time (minute)	With exenatide (mean ± SD)	Without exenatide (mean ± SD)	*p*
Glucose	60	0.37 ± 0.25	0.50 ± 0.13	0.032
	120	0.20 ± 0.23	0.49 ± 0.16	<0.001
	180	−0.17 ± 0.24	0.36 ± 0.17	0.005

Insulin	60	2.12 ± 0.98	2.74 ± 1.45	0.08
	120	3.88 ± 2.50	5.93 ± 2.62	0.001
	180	1.50 ± 1.21	2.21 ± 0.90	0.027

Ghrelin	60	−0.07 ± 0.13	0.10 ± 0.33	0.074
	120	−0.07 ± 0.20	−0.04 ± 0.51	0.221
	180	−0.20 ± 0.15	0.10 ± 0.29	0.001

SD: standard deviation.

**Table 4:** Comparison of total AUC values following mixed meal test with and without exenatide usage.

	With exenatide (mean ± SD)	Without exenatide (mean ± SD)	*p*
Glucose total AUC (mg·min/dL)	31403.6 ± 4696.4	37742.1 ± 4170.8	0.001
Insulin total AUC (*µ*g·min/mL)	8172.4 ± 3137.5	10502.1 ± 3881.2	0.004
Ghrelin total AUC (pg·min/mL)	48021.4 ± 8991.7	52305.0 ± 12429.8	0.035

AUC: area under the curve.
